# Supplementary material for: Sex Determination in Two Species of Anuran Amphibians by Magnetic Resonance Imaging and Ultrasound Techniques
Source: Animals (Basel). 2020 Nov 18;10(11):2142. doi: 10.3390/ani10112142 (PMC7698606; doi:10.3390/ani10112142)
Supplement: Supplementary file 1 [file animals-10-02142-s001.pdf]

SUPPLEMENTARY MATERIAL

(5 pages)

Manuscript entitled: Sex determination in two species of anuran amphibians by magnetic resonance imaging and ultrasound techniques

1) **Figure S1 - MRI identification of structures contained in the coelomic cavity.**

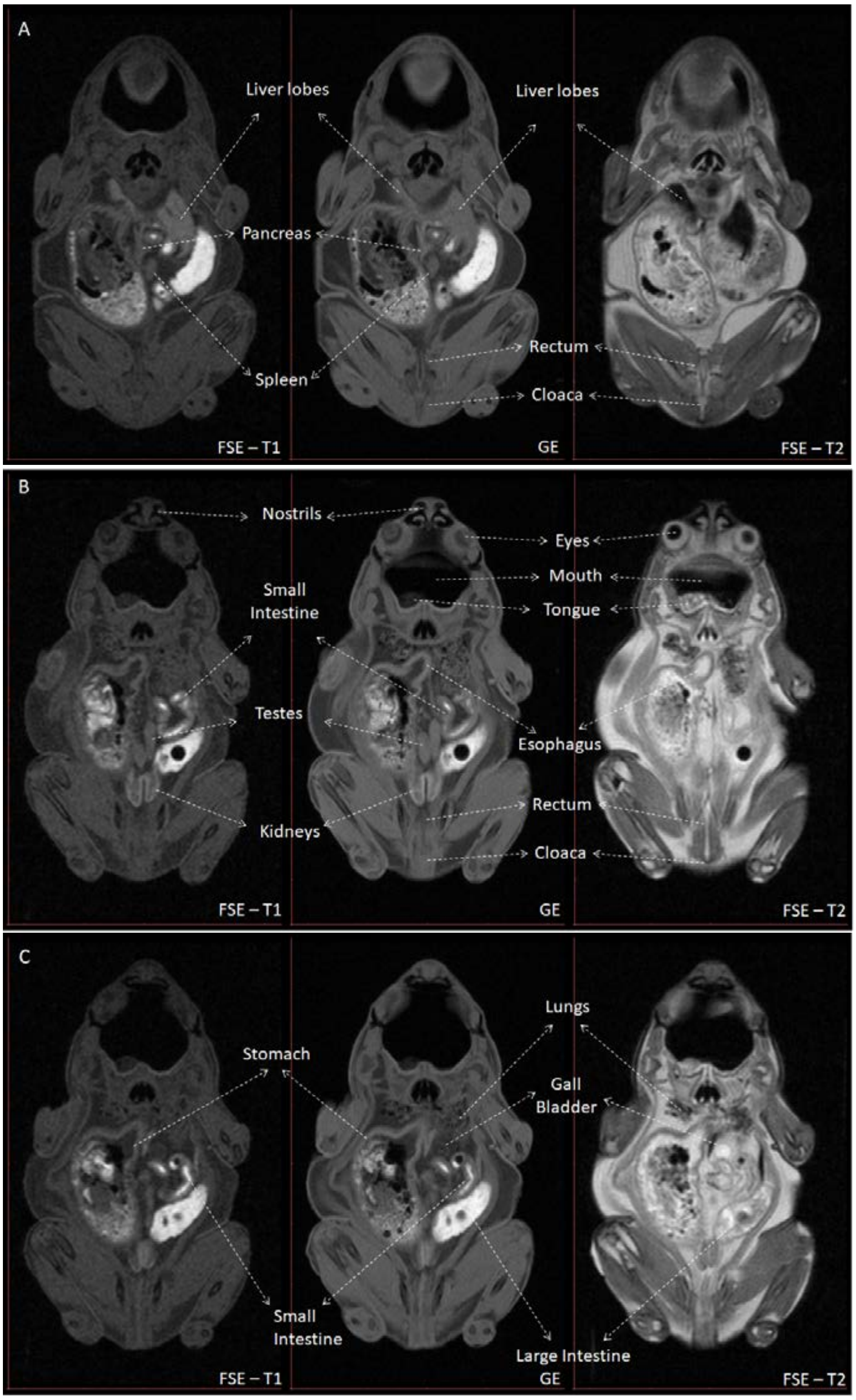

Figure S1 shows the main structures identified in the coelomic cavity in *Kaloula pluchra* frog. Three different slices of the MRI experiment were selected (A, B, C). Three different MRI weightings (Fast Spin Echo T1-weighted, Gradient Echo and FSE T2-weighted) are shown for each slice.

2) Graph S1. MRI - Volumetric quantification of testes and kidneys.

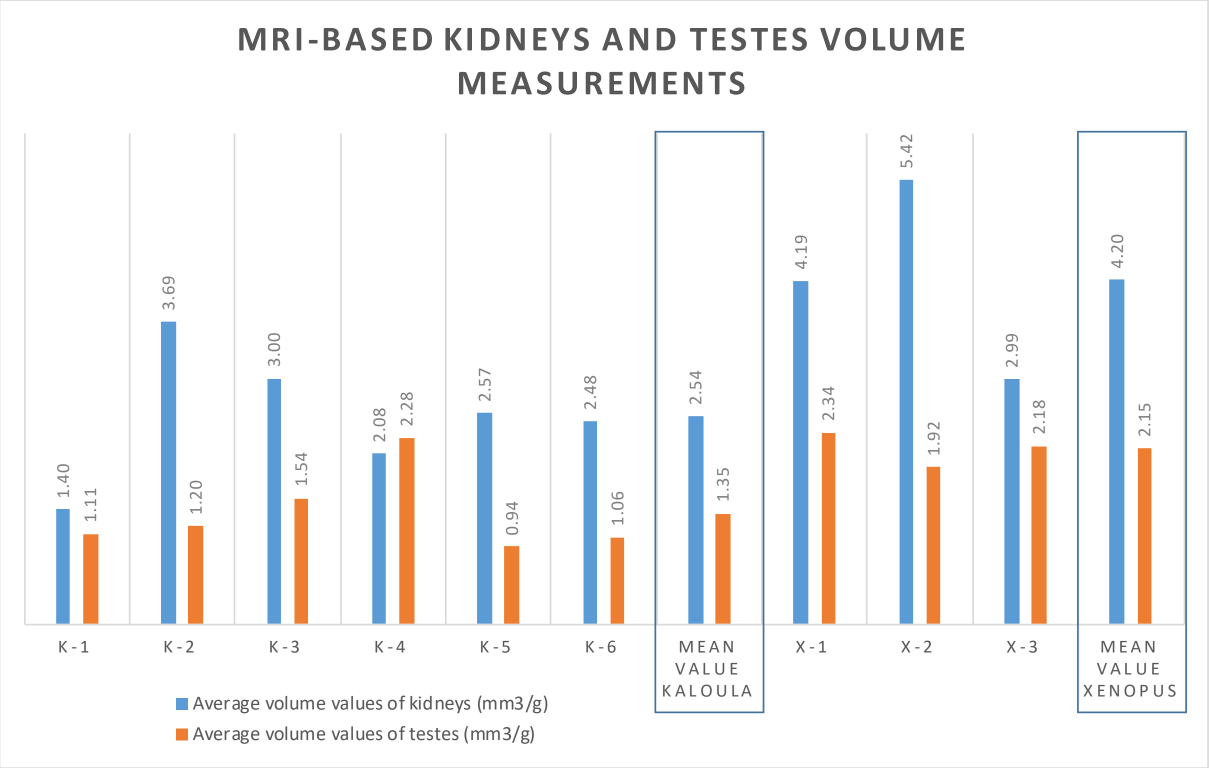

Graph S1 shows the average volume values weighted by body mass (mm^3/g) of kidneys and testes measured in six *Kaloula pulchra* (K-1 to K-6) and three *Xenopus laevis* anurans (X-1 to X-3). The graph is intended to show the possibility of obtaining quantitative information through the MRI study. This fact allows to contribute to the phenotyping between different species. As shown in the graph, regardless of the amphibian weight, the mean values for body-mass-weighted volume of kidneys and testis were considerably higher in *Xenopus laevis* anurans.

3) Figure S2 - Additional sex-gender system structures identified by MRI.

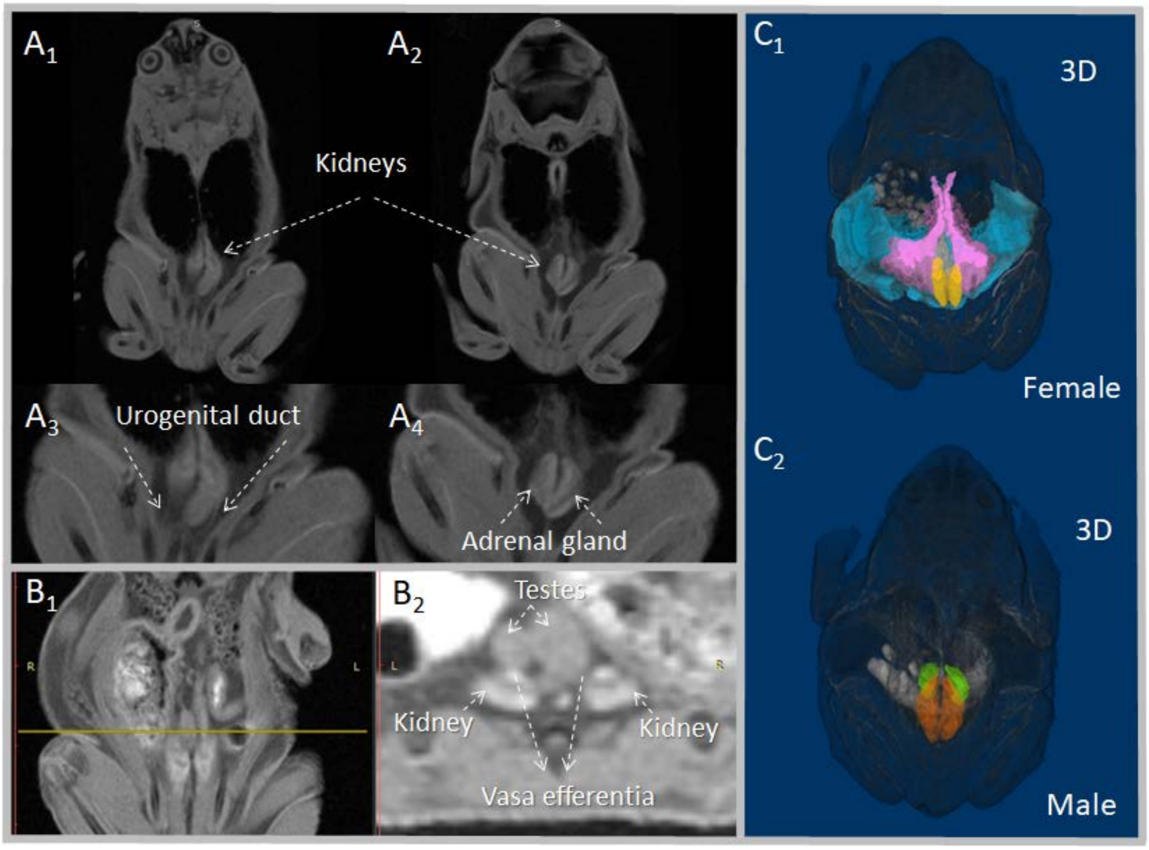

Figure S2. Composition shows additional sex-gender system structures identified by MRI (A and B) as well as the 3D visualization of the main organs of the male and female reproductive system in *Kaloula pulchra*. Figures A and B show the urogenital ducts, the adrenal glands and the vasa efferentia that connects testes with the urogenital duct, identified in a *Kaloula pulchra* male. Fig. C1 illustrates the 3D view of kidneys (yellow), oviduct (pink) and ovaries with follicles (sky blue) while Fig. C2 remarks testes (green) and kidneys (orange).

4) **Figure S3 - MRI identification of reproductive system disorders.**

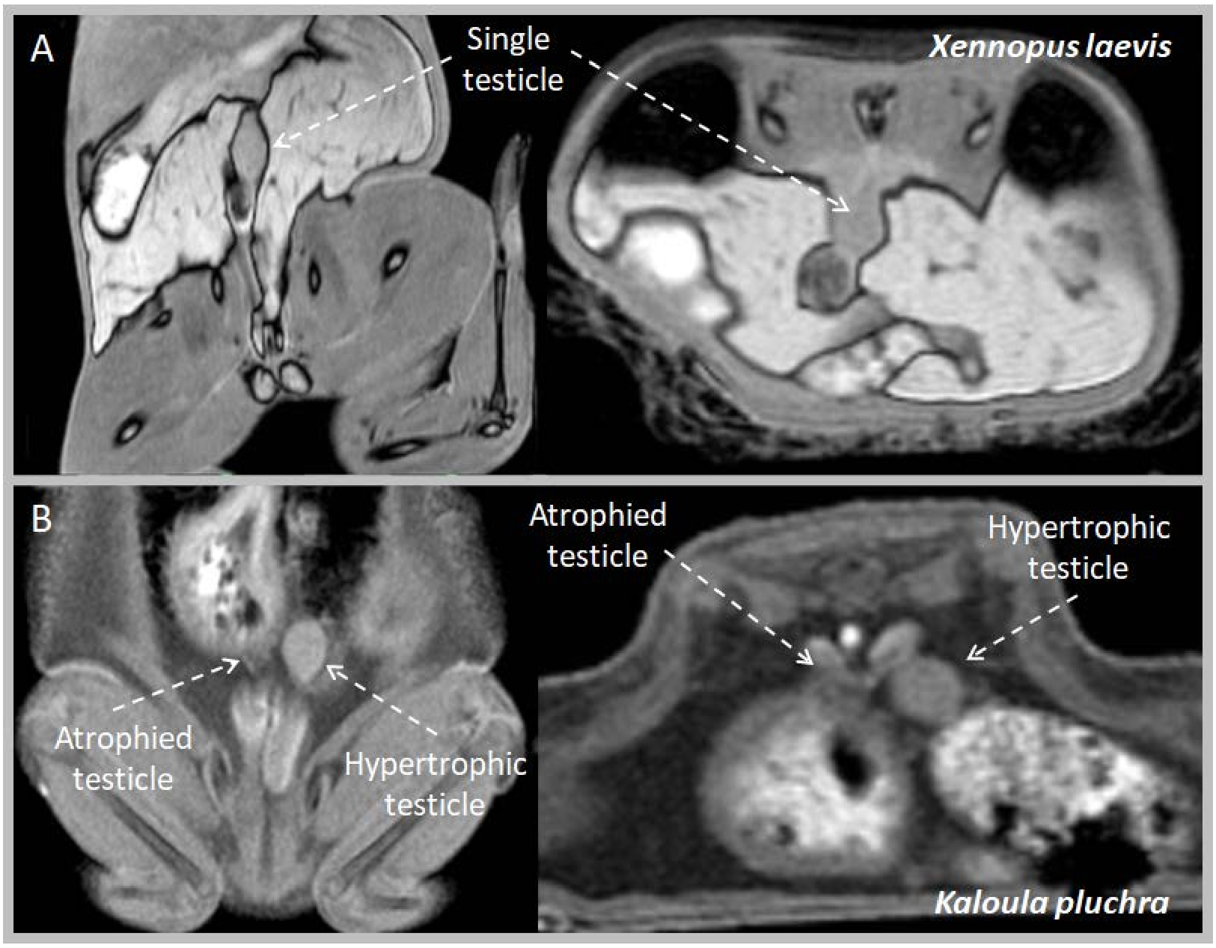

Figure S3 represents anomalies observed in the reproductive system of anurans during the MRI study. Fig. S3A shows a case in which only one testicle was observed in a *Xenopus laevis* male. Fig. S3B displays a *Kaloula pulchra* male with asymmetric and abnormally shaped testicles with atrophic appearance of the left testis and hypertrophic appearance of the right testis. Fig. S3A and Fig.S3B are clear examples of the additional information that MRI provides during a rapid study of gender determination.
